# Interpreting the Influence of Using Blood Donor Residual Samples for SARS-CoV-2 Seroprevalence Studies in Japan: Cross-Sectional Survey Study

**DOI:** 10.2196/60467

**Published:** 2025-02-10

**Authors:** Ryo Kinoshita, Sho Miyamoto, Tadaki Suzuki, Motoi Suzuki, Daisuke Yoneoka

**Affiliations:** 1 Center for Surveillance, Immunization, and Epidemiologic Research, National Institute of Infectious Diseases, 1-23-1 Toyama, Shinjuku-Ku, Tokyo 162-0052 Japan, Tokyo, Japan, 81 3-5285-1111; 2 Department of Pathology, National Institute of Infectious Diseases, Tokyo, Japan

**Keywords:** SARS-CoV-2, COVID-19, seroprevalence, blood donor, selection bias, healthy donor effect, coronavirus, pandemic, Japan, cross-sectional study, residual blood, epidemiology, blood donation, web-based, logistic regression, social economic, comorbidity, COVID-19 vaccination, public health

## Abstract

**Background:** Residual blood donor samples are commonly used in SARS-CoV-2 seroepidemiological studies; however their use may introduce bias due to the healthy donor effect, wherein blood donors are generally healthier than the general population. This potential bias is critical for accurately interpreting seroepidemiological data, as blood donors might not fully represent broader population-level infection rates.

**Objective:** This study aims to assess the potential bias in SARS-CoV-2 seroprevalence estimates derived from blood donor samples in Japan by examining the association between blood donation history and COVID-19 diagnosis. By quantifying the healthy donor effect, we seek to refine the interpretation of SARS-CoV-2 seroepidemiological studies using residual blood donor samples.

**Methods:** We conducted a web-based survey from December 14 to 28, 2023, recruiting 10,781 Japanese residents aged 16‐69, stratified by demographic factors to match national representation. Participants provided information on demographics, socioeconomic status, COVID-19 vaccination history, comorbidities, and blood donation experience. A logistic regression model adjusting for confounders such as age, sex, education, occupation, comorbidities, and vaccination status, was used to estimate the odds of COVID-19 diagnosis among blood donors compared to nondonors.

**Results:** Of the 10,781 participants, 3583 (33.2%) reported a history of COVID-19 diagnosis, and 5015 (46.5%) indicated they had donated blood at least once in their lifetime, and 1128 (10.5%) donated within the last year. Blood donors had mean of 13.5 (SD 43.6) donations and were older, with a mean age of 46.4 (SD 13.9) years, compared to 38.5 (SD 14.1) years for nondonors. Among blood donors, 39.9% had comorbidities (95% CI 38.5‐41.2) compared to 27.9% (95% CI 26.7‐29.0) of nondonors. Blood donors had 1.62 (95% CI: 1.48‐1.78) times higher odds of COVID-19 diagnosis compared to nondonors. The higher diagnosis rate among blood donors likely reflects increased social interactions and health-seeking behaviors, a phenomenon we refer to as the inverse healthy donor effect. This suggests that blood donor samples could overestimate SARS-CoV-2 seroprevalence when generalized to the broader Japanese population.

**Conclusions:** Higher COVID-19 diagnosis rates among blood donors may reflect increased community involvement and health-seeking behaviors, suggesting an inverse healthy donor effect. This pattern indicates that in terms of SARS-CoV-2 infection, blood donors might not represent the healthiest segment of the population. Consequently, seroprevalence studies using blood donor samples could overestimate SARS-CoV-2 infection rates in the general Japanese population. For more accurate public health surveillance, the development of statistical methods to adjust for this bias is recommended.

## Introduction

The COVID-19 pandemic, triggered by the rapid dissemination of SARS-CoV-2, has highlighted the critical need for robust epidemiological surveillance to decipher the virus’s transmission dynamics and shape effective public health strategies. As the pandemic intensified, the exponential increase in cases coupled with the presence of mild and asymptomatic infections strained traditional case-based surveillance systems. These systems primarily capture symptomatic cases seeking healthcare, leaving a substantial proportion of the infected population—particularly those with mild or asymptomatic infections—undetected. This complication led to the adoption of seroprevalence studies, which estimate the extent of infection by measuring the presence of nucleocapsid or spike antibodies in populations to provide a broader estimation of infection spread, inclusive of undiagnosed cases.

While seroprevalence studies are invaluable for assessing SARS-CoV-2 exposure at the population level, they are subject to inherent biases [1,2]. For example, antibody levels can wane over time, and variations in assay sensitivity or specificity may underestimate or overestimate the seroprevalence. When residual blood samples from blood donors are used, an additional potential bias known as the healthy donor effect is introduced [3]. This effect arises because blood donors are typically healthier than the general population [4-7], potentially resulting in an underestimation of true seroprevalence. Although blood donor–based SARS-CoV-2 seroepidemiological studies have been widely conducted across various countries [8-11], these studies have not fully accounted for the extent of this effect. Recognizing and addressing such bias is critical to improving the reliability of seroprevalence estimates and their use in public health planning.

Despite recognition of the healthy donor effect, its quantitative impact on SARS-CoV-2 seroprevalence estimates remains largely unexplored, introducing uncertainty in interpreting findings derived from blood donor samples [12]. Our study addresses this gap by focusing on the quantification of the healthy donor effect within Japan’s blood donor population, with the goal of improving the interpretation of SARS-CoV-2 seroprevalence estimates and providing a more accurate assessment of the population’s exposure to SARS-CoV-2.

## Methods

### Study Participants and Questionnaire

The study surveyed Japanese residents aged 16‐69 years—the age group eligible for blood donation in Japan—from all 47 prefectures who could respond in Japanese. The sample size was set at 10,829, based on an approximately 6.1% national blood donation rate reported in a 2022 Japanese Red Cross survey [13], with a 5% alpha error and 80% power. To ensure national representation, a quota-controlled sampling approach was applied, stratified by age, sex, and region, with quotas informed by the 2020 national census. Participants were recruited from Cross Marketing Inc.’s (Japan) panel of 5 million active and diverse members as of March 2024, who volunteered and were incentivized with point-based rewards redeemable for goods and services from affiliated businesses upon completion of the questionnaire. Panel members were accepted on a first-come-first-served basis until quotas for each age and prefecture category were met, ensuring coverage across all 47 prefectures, including both urban and rural populations. The survey was conducted from November 14 to 28, 2023, in alignment with a national seroepidemiological survey among blood donors in Japan. To ensure data completeness, respondents were requested to answer all questions.

The online questionnaire was developed based on a comprehensive literature review on blood donation and epidemiological surveys. For questions related to blood donation, we referenced validated items from governmental surveys [14]. The survey items were divided into three modules: (1) sociodemographic characteristics such as sex, age at the survey, residential prefecture, occupation, education level, and household income; (2) blood donation–related characteristics such as blood donation experience (yes or no) and the total number of blood donations; and (3) COVID-19-related and clinical questions such as the number and timing of prior COVID-19 infections, the number of COVID-19 vaccinations, and medical history. The definitions for COVID-19 vaccination, blood donation, and comorbidity are provided in Multimedia Appendix 1. A binary variable was created from medical history records to identify comorbidities that rendered participants ineligible for blood donation and medically unable to receive the COVID-19 vaccine (see Multimedia Appendix 2). These comorbidities included current treatment for periodontal disease or dental caries, a diagnosis of high blood pressure, diabetes, asthma, or chronic obstructive pulmonary disease, and a history of angina, myocardial infarction, stroke, or cancer.

### Statistical Analysis

First, sociodemographic data were tabulated separately for participants with and without blood donation experience to provide basic information on the study population. Continuous variables were analyzed using Mann-Whitney U test and categorical variables were analyzed using Fisher exact test. To estimate the association between COVID-19 infection and blood donation experience, a logistic regression model was used and the associated odds ratio was estimated. Based on a directed acyclic graph, these confounders were adjusted: age groups (16-29, 30-39, 40-49, 50-59, 60-69 years), sex, residential region, education level, occupation, number of blood donations, number of vaccinations, and comorbidities (Multimedia Appendix 3). As behavioral changes induced by vaccination are expected to differ by age group, an interaction term between age groups, and the number of vaccinations was also adjusted in the model. Further, to explore the model robustness, we conducted subgroup analyses using Firth logistic regression to account for potential bias due to small sample sizes or sparse data. Model performance was evaluated by calculating c-statistics, performing the Hosmer-Lemeshow test, and assessing multicollinearity using the adjusted generalized variance inflation factor (GVIF), where GVIF ^1/(2 x^ *^df^*^)^, with *df* representing the degrees of freedom. All data analyses were conducted using R software (version 4.3.2; R Foundation for Statistical Computing).

### Ethical Considerations

This study was reviewed and approved by the Institutional Review Board at the National Institute of Infectious Diseases (authorization no. 1579). Informed consent was obtained from all participants within the web survey, with only those providing consent allowed to proceed. All data used in the analysis were fully anonymized by Cross Marketing Inc. before being provided to the researchers. Participants received point-based rewards as compensation for survey completion, in line with institutional review board–approved ethical guidelines.

## Results

The main characteristics of the study participants are presented in Multimedia Appendix 4. A total of 10,781 participants were enrolled in Japan during the study period from December 14 to 28, 2023. Of these, 3583 (33.2%) had a history of COVID-19 diagnosis, and 5015 (46.5%) reported having donated blood at least once in their lifetime. Furthermore, 1128 (10.5%) had donated blood within the past year (Multimedia Appendix 5). Blood donors (defined as those with a history of blood donation) reported mean 13.5 (SD 43.6) donations. . Multimedia Appendix 6 shows the detailed distribution of blood donation frequency in our web survey. The mean age of blood donors was 46.4 (SD 13.9) years, whereas nondonors were younger, with a mean age of 38.5 (SD 14.1) years. Among blood donors, 39.9% (95%CI 38.5‐41.2) had comorbidities, compared to 27.9% (95% CI 26.7‐29.0) of nonblood donors (see Multimedia Appendix 7).

**Figure 1.**
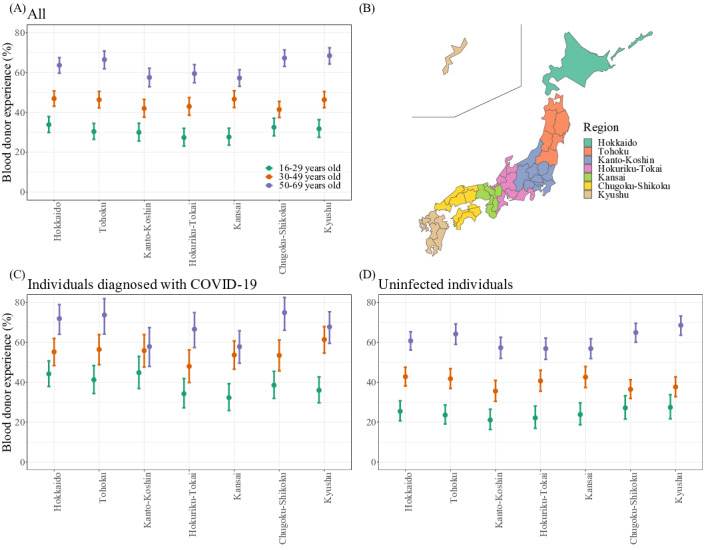
Proportion of blood donors and COVID-19 diagnosis status among Japanese residents aged 16‐69 years, based on a web-based survey conducted across all 47 prefectures in Japan from December 14 to 28, 2023. (A) Overall proportion of participants with blood donation experience. (B) Map showing geographic regions represented in the study. (C) Proportion of blood donors among individuals diagnosed with COVID-19. (D) Proportion of blood donors among uninfected (undiagnosed) individuals.

Figure 1A depicts the proportion of blood donors categorized by age group and geographic region in Japan. The corresponding geographic regions are illustrated in Figure 1B. In general, individuals who have been diagnosed with COVID-19 had a higher prevalence of prior blood donation compared to those without a history of diagnosis. The estimated results of the logistic regression model shown in Table 1. After excluding individuals with comorbidities that render them ineligible to donate blood or receive the COVID-19 vaccine, and after adjusting for potential confounders, we found that the odds of infection among blood donors were 1.62 times greater (95%CI 1.48‐1.78) than nondonors. Overall, the positive odds ratio was similar across most subgroup analysis with different stratifications, with most results being statistically significant (see Multimedia Appendix 8 ). Model validation resulted in a c-statistic of 0.66, a Hosmer-Lemeshow test value of *χ*^²^=6.78 (*P*=.56), and adjusted GVIF values below 5, with a mean of 1.34.

**Table 1.** Logistic regression results examining the association between COVID-19 diagnosis (outcome) and blood donor experience (main variable of interest) among Japanese residents. Odds ratios with 95% CI and *P* values are provided. Additional variables, including demographic, socioeconomic, and health-related factors, were included as adjustment variables based on a directed acyclic graph (DAG) to control for potential confounding. Vaccination status, household income, and blood donor frequency were included as continuous variables.

Variables	Odds Ratio (OR)	(95% CI)	*P* value
Age group (years)			
16‐29 (Ref)^a^	1.00	–^b^	–
30‐39	0.88	(0.67‐1.14)	.37
40‐49	0.59	(0.48‐0.74)	<.001
50‐59	0.59	(0.46‐0.77)	<.001
60‐69	0.55	(0.40‐0.77)	<.001
Vaccination status	1.05	(1.01-1.10)	.04
Sex			
Female (Ref)^a^	1.00	–	–
Male	0.90	(0.82‐0.99)	.05
Region			
Hokkaido	1.13	(0.97‐1.32)	.14
Tohoku	0.97	(0.82‐1.14)	.73
Kanto-Koshin (Ref)^a^	1.00	–	–
Hokuriku-Tokai	1.11	(0.94‐1.31)	.27
Kansai	1.28	(1.09‐1.50)	.004
Chugoku-Shikoku	1.07	(0.91‐1.26)	.47
Kyushu	1.39	(1.19‐1.62)	<.001
Highest level of education			
Middle school / High school	1.01	(0.92‐1.11)	.85
Junior. college / Vocational school / University (Ref)	1.00	–	–
Graduate school (Master / PhD)	0.72	(0.58‐0.89)	.004
Occupation			
Commerce	1.72	(1.44‐2.06)	<.001
Construction / Manufacturing / Transportation	1.75	(1.46‐2.09)	<.001
Education / Student	2.39	(1.96‐2.92)	<.001
Food / Beverage / Accommodation	1.51	(1.10‐2.06)	.01
Homemaker	1.88	(1.49‐2.37)	<.001
Information / Communication	1.92	(1.52‐2.44)	<.001
Medical / Social welfare	2.53	(2.05‐3.13)	<.001
Primary industries	1.75	(1.15‐2.65)	.01
Public servant	2.22	(1.77‐2.80)	<.001
Other	1.38	(1.10‐1.75)	.01
Unemployed (Ref)	1.00	–	–
Household Income	1.06	(1.04‐1.07)	<.001
Blood donor experience			
No (Ref)^a^	1.00	–	–
Yes	1.62	(1.48‐1.78)	<.001
Blood donor frequency	1.00	(1.00‐1.00)	.85
Comorbidity			
No (Ref)^a^	1.00	–	–
Yes	1.40	(1.28‐1.53)	<.001
Age group (years) * Vaccination Status			
Age group 16‐29 * Vaccination status (Ref)^a^	1.00	–	–
Age group 30‐39 * Vaccination status	0.93	(0.86‐1.01)	.12
Age group 40‐49 * Vaccination status	0.95	(0.89‐1.02)	.16
Age group 50‐59 * Vaccination status	0.87	(0.81‐0.94)	<.001
Age group 60‐69 * Vaccination status	0.86	(0.79‐0.93)	<.001

a Ref: Reference variable.

b –: Not available.

## Discussion

Our study showed a significant association between blood donation experience and COVID-19 diagnosis. However, the diagnosis rates did not increase with the frequency of donations, suggesting that the act of donating blood itself did not contribute to the transmission risk. Instead the higher incidence of COVID-19 among donors may be attributed to increased social interactions and greater health awareness, which could be characterized as an ‘inverse’ healthy donor effect [15]. Donors’ community involvement could raise exposure risks, while their health vigilance could lead to more frequent SARS-CoV-2 testing, leading to higher COVID-19 diagnosis. Further studies are needed to understand the potential underlying mechanism behind this association.

We found that using blood donor samples could potentially bias seroprevalence estimates in Japan. The observed positive association suggests that these samples may overestimate the actual rates compared to the general population. Previous efforts to adjust for demographic variances, such as age, gender, and prefecture, have not completely resolved the bias inherent in using blood donor samples [11]. This highlights the need to measure the healthy donor effect for accurate SARS-CoV-2 seroprevalence interpretations and emphasizes the challenge of correctly determining incidence from donor data, underscoring the need for improved research methodologies. Development of a statistical method to adjust for this bias of blood donor samples in estimating the seroprevalence of the general population of Japan using blood donor samples remains an ongoing focus of our research group.

There are a few limitations to note. First, the identification of COVID-19 cases was based on clinical diagnoses rather than antibody-confirmed infection rates, suggesting that the study may reflect differences in health-seeking behavior rather than actual SARS-CoV-2 infection rates. Second, although we applied quota sampling based on demographic distributions from the national census to improve representation, the sample population may not fully represent the general population in Japan, as the study was conducted via a web-based survey. This approach may introduce potential selection bias, particularly excluding individuals without internet access or those less comfortable with technology. Third, the study relied on self-reported data, which may introduce recall bias or social desirability bias. Fourth, we may not have fully adjusted for residual confounding from variables that were not included in the survey. Fifth, the cross-sectional design has inherent limitations, including difficulty determining temporal relationships and tracking changes over time. Finally, our findings may be specific to the Japanese context and might not necessarily apply to other countries due to differences in demographics, healthcare systems, and social behavior. Further evaluation is needed to assess the external validity across different countries or regions.

In conclusion, our study has identified a potential bias in SARS-CoV-2 seroprevalence studies that use residual samples from blood donors. Quantifying the direction and magnitude of this bias is essential for accurately interpreting seroprevalence surveys, especially when these surveys are used to guide infectious disease surveillance.

## Supplementary material

10.2196/60467 Multimedia Appendix 1 Description of COVID-19 vaccination and blood donation-related variables included in the model.

10.2196/60467 Multimedia Appendix 2 Description of comorbidities that affect eligibility for blood donation in Japan, used to create the binary variable Comorbidity.

10.2196/60467 Multimedia Appendix 3 Directed acyclic graph (DAG) illustrating the relationship between blood donor experience (exposure) and COVID-19 infection (outcome), with other variables included as potential confounders.

10.2196/60467 Multimedia Appendix 4 Demographic, socioeconomic, and health characteristics of study participants in the web survey, categorized by COVID-19 diagnosis status (uninfected or infected) and blood donation status (nonblood donor or blood donor).

10.2196/60467 Multimedia Appendix 5 Comparison of blood donation rates within 1 year among the sampled population (circle points) and Japanese Red Cross Society data for 2022 (star points), categorized by age group and region. The figure displays blood donation proportions across different age groups (16–29, 30–49, 50–69 years) and regions, with whiskers representing 95% confidence intervals.

10.2196/60467 Multimedia Appendix 6 Frequency distribution of blood donation experience among web survey participants. (A) Number of blood donations over participants' entire lifetime. (B) Number of blood donations within the past year. The X-axis is presented on a logarithmic scale to better illustrate the range and variability in donation frequencies across the study population.

10.2196/60467 Multimedia Appendix 7 Proportion of comorbidities among participants by blood donor status (0 = nondonor, 1 = donor). Whiskers represent 95% confidence intervals.

10.2196/60467 Multimedia Appendix 8 Supplementary Table 4.

## Abbreviations

GVIF: generalized variance inflation factor
