# Supplementary material for: Interpreting the Influence of Using Blood Donor Residual Samples for SARS-CoV-2 Seroprevalence Studies in Japan: Cross-Sectional Survey Study
Source: JMIR Public Health Surveill. 2025 Feb 10;11:e60467. doi: 10.2196/60467 (PMC11833190; doi:10.2196/60467)
Supplement: Multimedia Appendix 1 [file publichealth-v11-e60467-s001.docx]

Multimedia Appendix 1: Description of COVID-19 vaccination and blood donation-related variables included in the model.

| Variable | Definition |
| --- | --- |
| COVID-19 vaccine | 1 to ≤6 doses |
| Blood donor experience | 0 = No prior experience, 1 = Prior experience |
| Blood donor experience within 1 year | 0 = No donations within the last year, 1 = Donated within the last year |
| Blood donor frequency | Total number of lifetime donations |
| Blood donor frequency within 1 year | Number of donations in the past year |
| Comorbidity* | 0 = No comorbidity, 1 = Has comorbidity |

*See Multimedia Appendix 2 for the definition of comorbidity included in the survey
