# Supplementary material for: Interpreting the Influence of Using Blood Donor Residual Samples for SARS-CoV-2 Seroprevalence Studies in Japan: Cross-Sectional Survey Study
Source: JMIR Public Health Surveill. 2025 Feb 10;11:e60467. doi: 10.2196/60467 (PMC11833190; doi:10.2196/60467)
Supplement: Multimedia Appendix 2 [file publichealth-v11-e60467-s002.docx]

Multimedia Appendix 2: Description of comorbidities that affect eligibility for blood donation in Japan, used to create the binary variable *Comorbidity*.

| Comorbidity | Definition | Eligibility to Donate Blood in Japan |
| --- | --- | --- |
| COVID-19 vaccine | Unable to get vaccinated due to medical reasons | Depends on the underlying medical condition preventing vaccination |
| Hypertension | Currently diagnosed | Possible, if controlled without complications. Deferral if under certain treatments (e.g., blood pressure medication) |
| Diabetes | Currently diagnosed | Possible, if controlled without insulin or complications such as retinopathy or nephropathy |
| Asthma | Currently or previously diagnosed | Possible if no symptoms at the time of donation and well-managed |
| Bronchitis/Pneumonia | Currently or previously diagnosed | Deferral until complete recovery from active infection |
| Periodontal disease | Currently diagnosed and under treatment | For dental treatments with bleeding (including tartar removal), deferred for 3 days due to bacteremia risk |
| Dental cavities | Currently diagnosed and under treatment | For dental treatments with bleeding (including tartar removal), deferred for 3 days due to bacteremia risk |
| Angina pectoris | Currently or previously diagnosed | Permanent deferral due to heart condition severity |
| Myocardial infarction | Currently or previously diagnosed | Permanent deferral |
| Stroke | Currently or previously diagnosed | Permanent deferral |
| COPD (Chronic Obstructive Pulmonary Disease) | Currently diagnosed | Likely permanent deferral based on lung function impairment |
| Cancer/Malignant tumor | Currently or previously diagnosed | Permanent deferral for most cancers. Temporary deferral possible for non-metastatic skin cancers after complete treatment |
