# Supplementary material for: Interpreting the Influence of Using Blood Donor Residual Samples for SARS-CoV-2 Seroprevalence Studies in Japan: Cross-Sectional Survey Study
Source: JMIR Public Health Surveill. 2025 Feb 10;11:e60467. doi: 10.2196/60467 (PMC11833190; doi:10.2196/60467)

Multimedia Appendix 3. Directed acyclic graph (DAG) illustrating the relationship between blood donor experience (exposure) and COVID-19 infection (outcome), with other variables included as potential confounders.


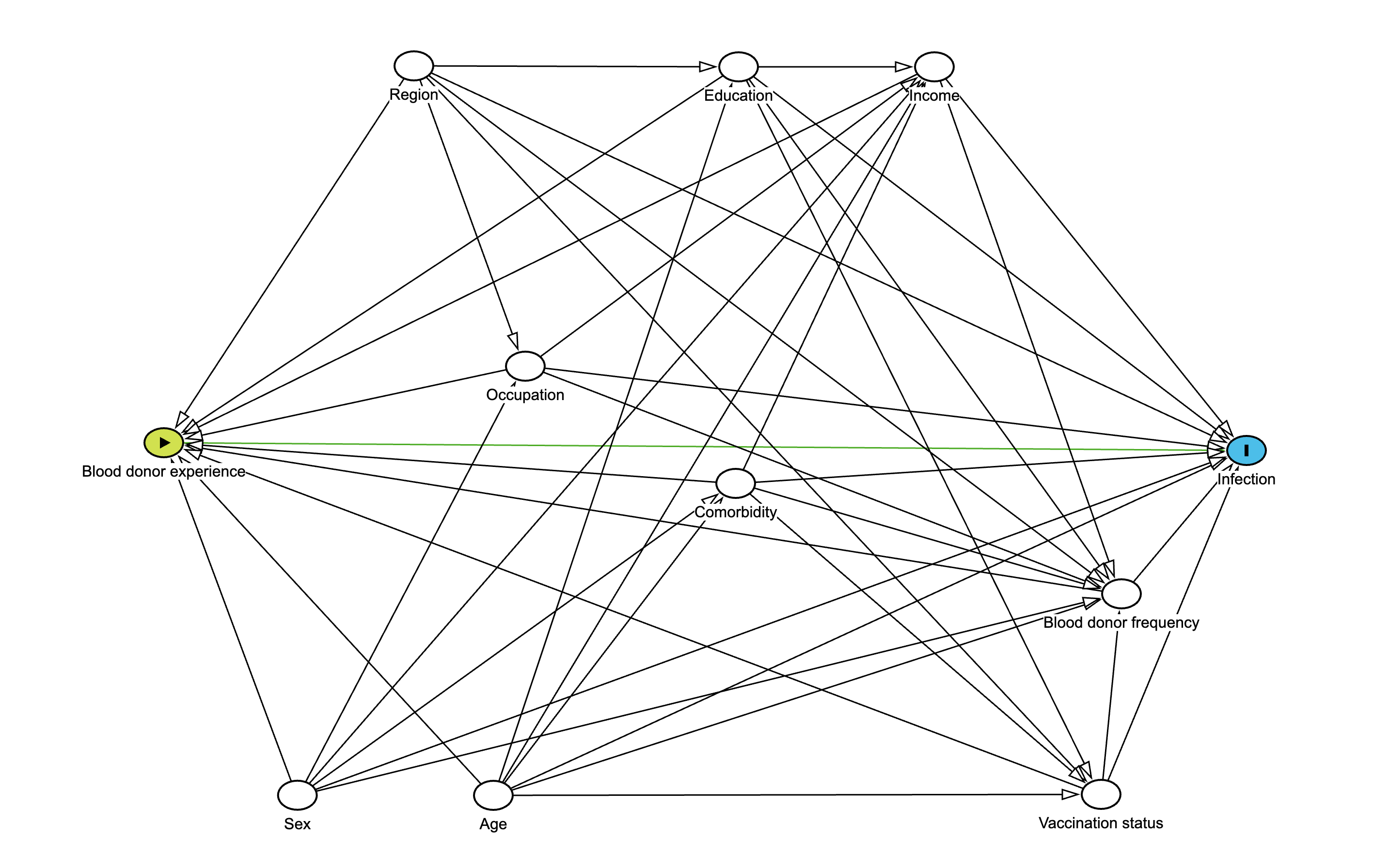

Supplement: Multimedia Appendix 3 [file publichealth-v11-e60467-s003.docx]
