# Supplementary material for: Interpreting the Influence of Using Blood Donor Residual Samples for SARS-CoV-2 Seroprevalence Studies in Japan: Cross-Sectional Survey Study
Source: JMIR Public Health Surveill. 2025 Feb 10;11:e60467. doi: 10.2196/60467 (PMC11833190; doi:10.2196/60467)
Supplement: Multimedia Appendix 4 [file publichealth-v11-e60467-s004.docx]

Multimedia Appendix 4. Demographic, socioeconomic, and health characteristics of study participants in the web survey, categorized by COVID-19 diagnosis status (uninfected/infected) and blood donation status (non-blood donor/blood donor).

|  | Overall | Uninfected | Infected | *P*-value |  |  | Non-blood donor | Blood donor | *P*-value |
| --- | --- | --- | --- | --- | --- | --- | --- | --- | --- |
| N | 10781 | 7198 | 3583 |  |  |  | 5766 | 5015 |  |
| Age group, N (%) |  |  |  |  |  |  |  |  |  |
| 16-29 | 3238 (30.0) | 1835 (25.5) | 1403 (39.2) (39.2) | <.001 |  |  | 2246 (39.0) | 992 (19.8) | <.001 |
| 30-39 | 1244 (11.5) | 793 (11.0) | 451 (12.6) |  |  |  | 744 (12.9) | 500 (10.0) |  |
| 40-49 | 2733 (25.4) | 1895 (26.3) | 838 (23.4) |  |  |  | 1452 (25.2) | 1281 (25.5) |  |
| 50-59 | 1994 (18.5) | 1452 (20.2) | 542 (15.1) |  |  |  | 796 (13.8) | 1198 (23.9) |  |
| 60-69 | 1572 (14.6) | 1223 (17.0) | 349 (9.7) |  |  |  | 528 ( 9.2) | 1044 (20.8) |  |
| Vaccination status, N (%) |  |  |  |  |  |  |  |  |  |
| 0 | 1867 (17.3) | 1399 (19.4) | 468 (13.1) | <.001 |  |  | 1323 (22.9) | 544 (10.8) | <.001 |
| 1 | 140 (1.3) | 59 (0.8) | 81 (2.3) |  |  |  | 66 (1.1) | 74 (1.5) |  |
| 2 | 1492 (13.8) | 891 (12.4) | 601 (16.8) |  |  |  | 909 (15.8) | 583 (11.6) |  |
| 3 | 2904 (26.9) | 1717 (23.9) | 1187 (33.1) |  |  |  | 1563 (27.1) | 1341 (26.7) |  |
| 4 | 2160 (20.0) | 1452 (20.2) | 708 (19.8) |  |  |  | 1037 (18.0) | 1123 (22.4) |  |
| 5 | 1194 (11.1) | 882 (12.3) | 312 (8.7) |  |  |  | 524 (9.1) | 670 (13.4) |  |
| 6- | 1024 (9.5) | 798 (11.1) | 226 (6.3) |  |  |  | 344 (6.0) | 680 (13.6) |  |
| Blood donor experience, N (%) |  |  |  |  |  |  |  |  |  |
| No | 5766 (53.5) | 4038 (56.1) | 1728 (48.2) | <.001 |  |  | NA | NA |  |
| Yes | 5015 (46.5) | 3160 (43.9) | 1855 (51.8) |  |  |  | NA | NA |  |
| Blood donor frequency, mean (SD) | 6.3 (30.5) | 6.2 (24.7) | 6.4 (39.7) | 0.716 |  |  | 0.0 (0.0) | 13.5 (43.6) | <.001 |
| Infection, N (%) |  |  |  |  |  |  |  |  |  |
| No | 7198 (66.8) | NA | NA |  |  |  | 3034 (70.5) | 2016 (64.1) | <.001 |
| Yes | 3583 (33.2) | NA | NA |  |  |  | 1267 (29.5) | 1130 (35.9) |  |
| Income (Japanese Yen), N (%) |  |  |  |  |  |  |  |  |  |
| -1,000,000 | 1137 (10.5) | 840 (11.7) | 297 (8.3) | <.001 |  |  | 774 (13.4) | 363 (7.2) | <.001 |
| 1,000,000-2,000,000 | 865 (8.0) | 667 (9.3) | 198 (5.5) |  |  |  | 518 (9.0) | 347 (6.9) |  |
| 2,000,000-3,000,000 | 1266 (11.7) | 910 (12.6) | 356 ( 9.9) |  |  |  | 731 (12.7) | 535 (10.7) |  |
| 3,000,000-4,000,000 | 1470 (13.6) | 990 (13.8) | 480 (13.4) |  |  |  | 790 (13.7) | 680 (13.6) |  |
| 4,000,000-5,000,000 | 1350 (12.5) | 858 (11.9) | 492 (13.7) |  |  |  | 708 (12.3) | 642 (12.8) |  |
| 5,000,000-6,000,000 | 1097 (10.2) | 672 (9.3) | 425 (11.9) |  |  |  | 540 (9.4) | 557 (11.1) |  |
| 6,000,000-7,000,000 | 926 (8.6) | 586 (8.1) | 340 (9.5) |  |  |  | 446 (7.7) | 480 (9.6) |  |
| 7,000,000-8,000,000 | 737 (6.8) | 436 (6.1) | 301 (8.4) |  |  |  | 352 (6.1) | 385 (7.7) |  |
| 8,000,000-9,000,000 | 503 (4.7) | 306 (4.3) | 197 (5.5) |  |  |  | 231 (4.0) | 272 (5.4) |  |
| 9,000,000-10,000,000 | 461 (4.3) | 293 (4.1) | 168 (4.7) |  |  |  | 220 (3.8) | 241 (4.8) |  |
| 10,000,000- | 969 (9.0) | 640 (8.9) | 329 (9.2) |  |  |  | 456 (7.9) | 513 (10.2) |  |
| Sex, N (%) |  |  |  |  |  |  |  |  |  |
| Female | 3473 (32.2) | 2219 (30.8) | 1254 (35.0) | <.001 |  |  | 2182 (37.8) | 1291 (25.7) | <.001 |
| Male | 7308 (67.8) | 4979 (69.2) | 2329 (65.0) |  |  |  | 3584 (62.2) | 3724 (74.3) |  |
| Region, N (%) |  |  |  |  |  |  |  |  |  |
| Hokkaido | 1815 (16.8) | 1204 (16.7) | 611 (17.1) | <.001 |  |  | 935 (16.2) | 880 (17.5) | .004 |
| Tohoku | 1554 (14.4) | 1073 (14.9) | 481 (13.4) |  |  |  | 827 (14.3) | 727 (14.5) |  |
| Kanto-Koshin | 1491 (13.8) | 1029 (14.3) | 462 (12.9) |  |  |  | 835 (14.5) | 656 (13.1) |  |
| Hokuriku-Tokai | 1248 (11.6) | 849 (11.8) | 399 (11.1) |  |  |  | 706 (12.2) | 542 (10.8) |  |
| Kansai | 1559 (14.5) | 1008 (14.0) | 551 (15.4) |  |  |  | 858 (14.9) | 701 (14.0) |  |
| Chugoku-Shikoku | 1557 (14.4) | 1061 (14.7) | 496 (13.8) |  |  |  | 818 (14.2) | 739 (14.7) |  |
| Kyushu | 1557 (14.4) | 974 (13.5) | 583 (16.3) |  |  |  | 787 (13.6) | 770 (15.4) |  |
| Highest level of education, N (%) |  |  |  |  |  |  |  |  |  |
| Middle school / High school | 4336 (40.2) | 2895 (40.2) | 1441 (40.2) | .130 |  |  | 2599 (45.1) | 1737 (34.6) | <.001 |
| Jr. college / Vocational school / University | 5941 (55.1) | 3946 (54.8) | 1995 (55.7) |  |  |  | 2936 (50.9) | 3005 (59.9) |  |
| Graduate school (Master / PhD) | 504 (4.7) | 357 (5.0) | 147 (4.1) |  |  |  | 231 (4.0) | 273 (5.4) |  |
| Occupation, N (%) |  |  |  |  |  |  |  |  |  |
| Commerce | 2190 (20.3) | 1495 (20.8) | 695 (19.4) | <.001 |  |  | 1190 (20.6) | 1000 (19.9) | <.001 |
| Construction / Manufacturing / Transportation | 2451 (22.7) | 1649 (22.9) | 802 (22.4) |  |  |  | 1147 (19.9) | 1304 (26.0) |  |
| Education / Student | 1111 (10.3) | 612 (8.5) | 499 (13.9) |  |  |  | 770 (13.4) | 341 (6.8) |  |
| Food / Beverage / Accommodation | 260 (2.4) | 183 (2.5) | 77 (2.1) |  |  |  | 155 (2.7) | 105 (2.1) |  |
| Homemaker | 706 (6.5) | 475 (6.6) | 231 (6.4) |  |  |  | 425 (7.4) | 281 (5.6) |  |
| Information / Communication | 568 (5.3) | 368 (5.1) | 200 (5.6) |  |  |  | 257 (4.5) | 311 (6.2) |  |
| Medical / Social welfare | 852 (7.9) | 476 (6.6) | 376 (10.5) |  |  |  | 420 (7.3) | 432 (8.6) |  |
| Primary industries | 118 (1.1) | 76 (1.1) | 42 (1.2) |  |  |  | 54 (0.9) | 64 (1.3) |  |
| Public servant | 629 (5.8) | 369 (5.1) | 260 (7.3) |  |  |  | 209 (3.6) | 420 (8.4) |  |
| Other | 633 (5.9) | 460 (6.4) | 173 (4.8) |  |  |  | 383 (6.6) | 250 (5.0) |  |
| Unemployed | 1263 (11.7) | 1035 (14.4) | 228 (6.4) |  |  |  | 756 (13.1) | 507 (10.1) |  |
| Comorbidity, mean (SD) | 0.3 (0.5) | 0.3 (0.5) | 0.4 (0.5) | .001 |  |  | 0.3 (0.4) | 0.4 (0.5) | <.001 |
