# Supplementary material for: Interpreting the Influence of Using Blood Donor Residual Samples for SARS-CoV-2 Seroprevalence Studies in Japan: Cross-Sectional Survey Study
Source: JMIR Public Health Surveill. 2025 Feb 10;11:e60467. doi: 10.2196/60467 (PMC11833190; doi:10.2196/60467)
Supplement: Multimedia Appendix 5 [file publichealth-v11-e60467-s005.docx]

Multimedia Appendix 5. Comparison of blood donation rates within 1 year among the sampled population (circle points) and Japanese Red Cross Society data for 2022 (star points), categorized by age group and region. The figure displays blood donation proportions across different age groups (16–29, 30–49, 50–69) and regions, with whiskers representing 95% confidence intervals.
