# Supplementary material for: Interpreting the Influence of Using Blood Donor Residual Samples for SARS-CoV-2 Seroprevalence Studies in Japan: Cross-Sectional Survey Study
Source: JMIR Public Health Surveill. 2025 Feb 10;11:e60467. doi: 10.2196/60467 (PMC11833190; doi:10.2196/60467)
Supplement: Multimedia Appendix 6 [file publichealth-v11-e60467-s006.docx]

Multimedia Appendix 6. Frequency distribution of blood donation experience among web survey participants. (A) Number of blood donations over participants' entire lifetime. (B) Number of blood donations within the past year. The x-axis is presented on a logarithmic scale to better illustrate the range and variability in donation frequencies across the study population.
