# Supplementary material for: Interpreting the Influence of Using Blood Donor Residual Samples for SARS-CoV-2 Seroprevalence Studies in Japan: Cross-Sectional Survey Study
Source: JMIR Public Health Surveill. 2025 Feb 10;11:e60467. doi: 10.2196/60467 (PMC11833190; doi:10.2196/60467)
Supplement: Multimedia Appendix 7 [file publichealth-v11-e60467-s007.docx]

Multimedia Appendix 7. Proportion of comorbidities among participants by blood donor status (0=nondonor, 1= donor). Whiskers represent 95% confidence intervals.
