# Supplementary material for: Interpreting the Influence of Using Blood Donor Residual Samples for SARS-CoV-2 Seroprevalence Studies in Japan: Cross-Sectional Survey Study
Source: JMIR Public Health Surveill. 2025 Feb 10;11:e60467. doi: 10.2196/60467 (PMC11833190; doi:10.2196/60467)
Supplement: Multimedia Appendix 8 [file publichealth-v11-e60467-s008.docx]

Multimedia Appendix 8. Subgroup analysis of the logistic regression between COVID-19 diagnosis and blood donor experience. The odds ratio for individuals with blood donor experience is presented for each subgroup, with those without blood donor experience as the reference group. Analysis was performed using Firth’s logistic regression.

| **Stratification** | **Odds Ratio (OR)** | **(95% CI)** | **p-value** |
| --- | --- | --- | --- |
| **Age group** |  |  |  |
| **16-29** | 1.85 | (1.57 - 2.17) | <0.001 |
| **30-39** | 2.02 | (1.56 - 2.62) | <0.001 |
| **40-49** | 1.67 | (1.40 - 1.99) | <0.001 |
| **50-59** | 1.29 | (1.04 - 1.61) | 0.022 |
| **60-69** | 1.14 | (0.88 - 1.50) | 0.326 |
| **Vaccination status** |  |  |  |
| **0** | 1.88 | (1.47 - 2.42) | <0.001 |
| **1** | 1.28 | (0.48 - 3.46) | 0.614 |
| **2** | 1.87 | (1.47 - 2.39) | <0.001 |
| **3** | 1.56 | (1.32 - 1.85) | <0.001 |
| **4** | 1.46 | (1.20 - 1.79) | <0.001 |
| **5** | 1.73 | (1.28 - 2.33) | <0.001 |
| **6** | 1.06 | (0.75 - 1.52) | 0.725 |
| **Sex** |  |  |  |
| **Female** | 1.68 | (1.50 - 1.88) | <0.001 |
| **Male** | 1.52 | (1.29 - 1.79) | <0.001 |
| **Region** |  |  |  |
| **Hokkaido** | 1.70 | (1.36 - 2.13) | <0.001 |
| **Tohoku** | 1.65 | (1.29 - 2.12) | <0.001 |
| **Kanto-Koshin** | 1.60 | (1.26 - 2.03) | <0.001 |
| **Hokuriku-Tokai** | 1.62 | (1.23 - 2.13) | <0.001 |
| **Kansai** | 1.39 | (1.11 - 1.76) | 0.005 |
| **Chugoku-Shikoku** | 1.66 | (1.29 - 2.14) | <0.001 |
| **Kyushu** | 1.66 | (1.31 - 2.12) | <0.001 |
| **Highest level of education** |  |  |  |
| **Middle school / High school** | 1.75 | (1.50 - 2.04) | <0.001 |
| **Jr. college / Vocational school / University** | 1.53 | (1.36 - 1.72) | <0.001 |
| **Graduate school (Master / PhD)** | 1.78 | (1.15 - 2.78) | 0.010 |
| **Occupation** |  |  |  |
| **Commerce** | 1.58 | (1.29 - 1.94) | <0.001 |
| **Construction / Manufacturing / Transportation** | 1.88 | (1.56 - 2.27) | <0.001 |
| **Education / Student** | 1.57 | (1.17 - 2.11) | 0.003 |
| **Food / Beverage / Accommodation** | 1.54 | (0.84 - 3.53) | 0.162 |
| **Homemaker** | 1.10 | (0.74 - 1.63) | 0.649 |
| **Information / Communication** | 1.25 | (0.86 - 1.83) | 0.250 |
| **Medical / Social welfare** | 1.23 | (0.91 - 1.67) | 0.182 |
| **Primary industries** | 3.15 | (1.15 - 9.34) | 0.025 |
| **Public servant** | 1.95 | (1.34 - 2.85) | <0.001 |
| **Other** | 2.21 | (1.48 - 3.31) | <0.001 |
| **Unemployed** | 1.36 | (0.97 - 1.90) | 0.073 |
| **Comorbidity** |  |  |  |
| **Yes** | 1.64 | (1.40 - 1.92) | <0.001 |
| **No** | 1.60 | (1.43 - 1.80) | <0.001 |
